# Efficient Spectrum Occupancy Prediction Exploiting Multidimensional Correlations through Composite 2D-LSTM Models †

**DOI:** 10.3390/s21010135

**Published:** 2020-12-28

**Authors:** Mehmet Ali Aygül, Mahmoud Nazzal, Mehmet İzzet Sağlam, Daniel Benevides da Costa, Hasan Fehmi Ateş, Hüseyin Arslan

**Affiliations:** 1 Department of Electrical and Electronics Engineering, Istanbul Medipol University, Istanbul 34810, Turkey; mahmoud.nazzal@ieee.org (M.N.); hfates@medipol.edu.tr (H.F.A.); arslan.usf@gmail.com (H.A.); 2 Department of Research & Development, Turkcell, Istanbul 34880, Turkey; izzet.saglam@turkcell.com.tr; 3 Department of Computer Engineering, Federal University of Ceará (UFC), Sobral 62010-560, Brazil; danielbcosta@ieee.org; 4 Department of Electrical Engineering, University of South Florida, Tampa, FL 33620, USA

**Keywords:** cognitive radio, deep learning, multidimensions, real-world spectrum measurement, spectrum occupancy prediction

## Abstract

In cognitive radio systems, identifying spectrum opportunities is fundamental to efficiently use the spectrum. Spectrum occupancy prediction is a convenient way of revealing opportunities based on previous occupancies. Studies have demonstrated that usage of the spectrum has a high correlation over multidimensions, which includes time, frequency, and space. Accordingly, recent literature uses tensor-based methods to exploit the multidimensional spectrum correlation. However, these methods share two main drawbacks. First, they are computationally complex. Second, they need to re-train the overall model when no information is received from any base station for any reason. Different than the existing works, this paper proposes a method for dividing the multidimensional correlation exploitation problem into a set of smaller sub-problems. This division is achieved through composite two-dimensional (2D)-long short-term memory (LSTM) models. Extensive experimental results reveal a high detection performance with more robustness and less complexity attained by the proposed method. The real-world measurements provided by one of the leading mobile network operators in Turkey validate these results.

## 1. Introduction

Accommodating exploding data traffic is among one of the biggest challenges for the communication systems in the fifth-generation (5G) and beyond. In 5G networks, data rates will be multiplied by ten compared to fourth-generation [1], and latency will go down to one millisecond or less [2]. Therefore, the ever-increasing demanding nature of higher rate communications causes an inherent gap with the scarcity of the available spectrum [3]. Cognitive radio (CR) is believed to be one of the key solutions to bridge this gap [4].

CR enables secondary users (SUs) to opportunistically use available spectrum bands (referred to as spectrum holes) unused by primary users (PUs) [5]. It is evident that this needs identification of spectrum usage states, in a process referred to as spectrum sensing. Spectrum prediction is an alternative to continuous spectrum sensing [6,7,8,9] where future occupancies are predicted based on previous occupancies. This alternative substantially saves the time, energy, and computation overheads required by continuous spectrum sensing [10].

There are various spectrum occupancy prediction methods [11]. Early works used classical statistical prediction methods to predict holes. Examples include predicting holes using an exponential moving average model-based method [12], an autoregressive model (ARM)-based method [13], and a Bayesian inference (BIF)-based method [14]. More recently, machine learning (ML)-based methods have been preferred for this problem such as shallow artificial neural networks [15] and wavelet neural networks [16].

Recent literature views that spectrum occupancy is a non-stationary process [17]. However, the aforementioned methods may not always be capable of addressing this issue. This incapability has more strongly emerged with the diverse user types and increased user mobility envisaged in 5G and beyond. In view of these challenges, the non-stationary hidden Markov method exploited the time-varying feature of PU behaviors [18] and deep learning (DL) methods have been proposed as an advanced spectrum prediction framework for addressing this non-stationarity.

Advanced DL techniques, namely, convolutional neural networks (CNN) and long-short term memory (LSTM) are promising to exploit correlation with long lags. Accordingly, the phase and amplitude difference of data was used to train CNN classifiers for detecting the presence of radar signals with high accuracy [19]. Spectral and temporal correlations were used with LSTM models [20]. Moreover, the spectrum in a frequency hopping communication was predicted by an LSTM network [21]. This work was subsequently extended using the Taguchi approach [10]. Furthermore, deep neural networks, LSTM, and CNN-based models were designed. Then, their capabilities in spectrum occupancy prediction were compared [22]. In our previous work [23], time and frequency correlations were exploited to predict spectrum occupancy over real-world measurements.

**Figure 1:**
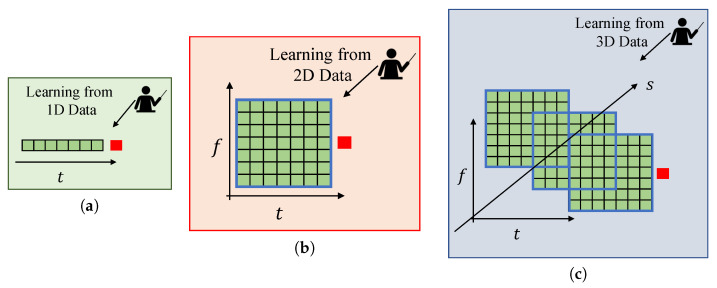
Spectrum occupancy prediction with (**a**) 1D data, (**b**) 2D data, (**c**) 3D data.

Although the aforementioned approaches have been useful in the analysis of numerous cases, they consider correlations only in time, space, and/or frequency domain. However, these dimensions do not provide a detailed analysis of the non-stationary characteristics and multidimensional attributes of the wireless signals [24]. Jointly exploiting multidimensional (time, frequency, and space) correlations provides a promising perspective for spectrum prediction, as illustrated in Figure 1. Recently, tensor analysis has been adopted as a framework to utilize multidimensional correlations for spectrum prediction. Along this line, the authors of [25] converted spectrum prediction into a third-order tensor completion problem. This approach achieved one-day-long predictions with a reasonable error margin. Another work considers merging CANDECOMP/PARAFAC tensor decomposition with LSTM for prediction [26]. Furthermore, multidimensional correlations were utilized jointly with convolutional long short-term memory (ConvLSTM) for a long-term temporal prediction [27].

Although tensor models provide a powerful and rich representation of a three-dimensional (3D) dataset, they share two common drawbacks. First is their high processing time [28,29]. Second, is that they assume 3D data can be provided at any time. However, sometimes, it is difficult to get information from all of the base stations (BS)s. For example, in the case of CR security threats (primary user emulation attack and jamming attack [30]), accurate information about spectrum occupancy cannot be provided from BSs. Also, in the case of a natural disaster, the information flow of some BSs can be cut. Therefore, such an assumption is not always valid or realistic.

To compensate for the aforementioned effects, more adaptive and flexible methods should be developed. In this regard, step-based methods [31], which divide the problem into smaller sub-problems, can be used. By the virtue of these methods, the complexity load in one model can be divided at the expense of temporal globality [32]. An attractive advantage of this setting is that it is not required to re-train the whole model once any element (BS) in the model fails to provide its information. Alternatively, the BS of which the information is missing temporarily is removed from the model, and only a simple end-classifier, which will give the last decision about spectrum occupancy, is re-trained.

In view of the above discussion, we extend a preliminary version of this work that uses two-dimensional (2D)-LSTM for spectrum occupancy prediction, appearing in [23]. In this extension, we exploit multidimensional correlations and propose the usage of composite 2D-LSTM models to divide the 3D spectrum occupancy prediction problem into smaller sub-problems. Extensive experimental results demonstrate that the proposed method can predict spectrum occupancies with less complexity and small performance loss compared to tensor-based methods. Furthermore, the performance of the proposed method is superior to the one-dimensional (1D) and 2D-based methods. Also, the proposed method does not require complete re-training with the absence of data from any BS. These results are validated over real-world spectrum measurements provided by one of the leading mobile network operators in Turkey. These measurements are made in two scenarios; city center and village, to reflect different user density scenarios. The *precision*, *recall*, and $F_{1}$-score performance metrics are used in this validation. Moreover, the training and testing execution time is used as a computational complexity metric.

*Organization*: This paper is organized as follows. Section 2 presents the system model and preliminaries. Section 3 details the proposed method. The data generation and experiments conducted to evaluate the performance of the proposed method are presented in Section 4 and Section 5, respectively. Finally, the paper is concluded in Section 6.

*Notation*: Scalars are represented by plain-faced letters. Vectors and matrices are denoted by bold-faced lower-case and bold-faced upper-case letters, respectively. In a vector x, the symbol $x_{i}$ denotes its i-th element. ⊙ denotes element-wise multiplication.

## 2. System Model and Preliminaries

**Figure 2:**
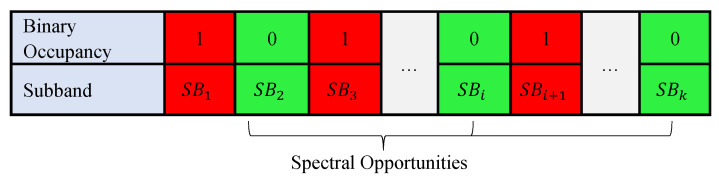
Spectrum subband (SB) occupancy modeling.

This work aims at predicting spectrum occupancy states over a given frequency range depending on the previous occupancies. For modeling the spectrum access, the heterogeneous spectrum access model [33] is adopted. This model is demonstrated in Figure 2. More specifically, the spectrum is split into *k* contiguous frequency subbands. In this model, the absence (presence) of a PU means signifies a hole (occupied spectrum). The following hypotheses ($H_{0}$ and $H_{1}$) formally states these cases.

(1) $$y=\left\{\begin{array}{cc} n, & H_{0}:\mathrm{there}\;\mathrm{is}\;\mathrm{no}\;\mathrm{PU}\\ Hx+n, & H_{1}:a\;\mathrm{PU}\;\mathrm{is}\;\mathrm{present}, \end{array}\right.$$

where x denotes PU transmitted signal, n represent the additive white Gaussian noise, channel matrix is represented by H and y denotes received signal. Furthermore, the works include state-of-the-art techniques used for spectrum occupancy and are briefly explained below.

### 2.1. Prediction with Autoregressive Model

The ARM [13] is a linear predictor and it works as follows

(2) $${\hat{s}}_{t}=\overset{r}{\sum_{i=1}}\varphi_{i}y_{t-i}+\omega_{t},$$

where *r* represents the model order, $\varphi_{i}$, i=1,2,⋯,r, is the model parameter, ${\hat{s}}_{t}$ and $\omega_{t}$ denote the predicted state at a future time instant *t* and white noise at time *t*, respectively, and $y_{t-i}$ is the observation at time instant t−i.

Predicting future states requires tuning the ARM parameters first. This can be achieved with several methods such as the Yule–Walker equations or maximum likelihood estimation. Once these parameters are tuned, they are used along with the historical values to predict the future states ${\hat{s}}_{t}$.

### 2.2. Prediction with Bayesian-Inference

BIF is a prediction method that uses the Bayes rule to update the probability distribution of a hypothesis when additional evidence data are learned. In this prediction method, a posterior probability distribution P(s|y) is derived by a CR user according to a Bayes rule as follows

(3) $$P\left(s|y\right)=\frac{P(y|s)\;\cdot \;P(s)}{P(y)},$$

where *s* denotes the spectrum occupancy. Afterward, the upcoming data are predicted by the derived posterior probability with the Bayes rule.

### 2.3. Prediction with Long Short-Term Memory

A more accurate data representation can be acquired by the use of multiple hidden layers in the deep architectures of DL models. The usage of multiple layers enables the model to magnify the intrinsic distinctive data features while suppressing the irrelevant information at each layer [34]. Thus, a primary advantage of DL is that it works directly on raw data. This means alleviating the human effort needed in any feature crafting/engineering. LSTM is an artificial recurrent neural network and it can be used as a DL model. This DL model is well-suited for handling grid-like data either in one, two, or multiple dimensions.

LSTM models have a memory block, which includes cells and gates. This memory block makes the model capable to use long short-term dependencies [35]. The gates are categorized under three parts according to their practical functionalities. These are input gates, forget gates, and output gates. Their transition equations are shown as follows

(4) $$\begin{array}{cc} & i_{t}=\sigma_{g}\left(W_{i}y_{t}+U_{i}h_{t-1}+b_{i}\right), \end{array}$$

(5) $$\begin{array}{cc} & f_{t}=\sigma_{g}\left(W_{f}y_{t}+U_{f}h_{t-1}+b_{f}\right), \end{array}$$

(6) $$\begin{array}{cc} & o_{t}=\sigma_{g}\left(W_{o}y_{t}+U_{o}h_{t-1}+b_{o}\right), \end{array}$$

(7) $$\begin{array}{cc} & {\tilde{c}}_{t}=\sigma_{c}\left(W_{c}y_{t}+U_{c}h_{t-1}+b_{c}\right), \end{array}$$

(8) $$\begin{array}{cc} & c_{t}=i_{t}⊙{\tilde{c}}_{t}+f_{t}⊙c_{t-1}, \end{array}$$

(9) $$\begin{array}{cc} & h_{t}=o{}_{t}⊙\sigma_{h}\left(c_{t}\right), \end{array}$$

where $i_{t}$, $f_{t}$, and $o_{t}$ represents input, forget, and output gate’s activation vectors, respectively and $y_{t}$ represents input vector at time *t*, $h_{t}$ represents hidden layer at *t* time step, ${\tilde{c}}_{t}$ represents cell input activation vector, $c_{t}$ denotes cell state vector, $\sigma_{g}$, $\sigma_{c}$, and $\sigma_{h}$ denote sigmoid function, hyperbolic tangent function, and hyperbolic tangent function, respectively, and biases, recurrent connections, and weights are denoted by b, U, W, respectively.

The above equations show that an input gate controls how a new value flows into the memory. On the other hand, how much of the past information to keep in the memory is decided by a forget gate and an output gate determines the weighting of the values to be used for computing the output activation of the block. Finally, the model can learn how to represent information over multiple time scales since the values of the gating variables vary for each vector element [36].

### 2.4. Prediction with Convolutional Long Short-Term Memory

ConvLSTM is a type of LSTM cell. More specifically, convolution takes place within the LSTM cell, and matrix multiplication is replaced with the convolution operation. The application of convolution allows capturing the spatial features from the image or data grid. The convolutional structures can be observed in ConvLSTM in both the input-to-state transition and state-to-state transition. It has been applied to activation from previous timestamps and input of the current timestamp. Further details can be found in [37].

## 3. The Proposed Method for Spectrum Occupancy Prediction Exploiting Time, Frequency, and Space Correlations

### 3.1. Motivations for Time, Frequency, and Space Correlation Exploitation and Problem Sub-Division

**Figure 3:**
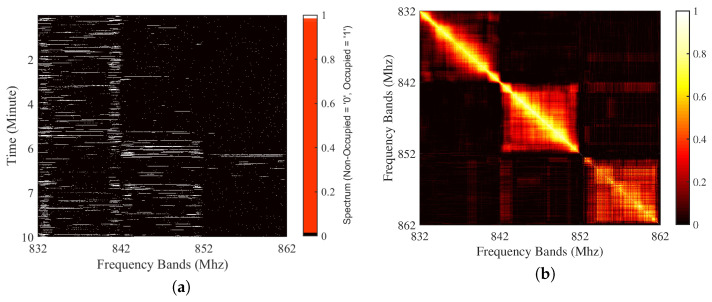
An example spectrum’s (**a**) occupancy distributions and (**b**) correlation.

Time series prediction depends on correlation over time. Similarly, the inherently existing frequency and space correlation can be exploited. The following test quantifies this correlation across a time-frequency grid. First, a day-long record of spectrum measurements has been obtained according to the measurement setup detailed in [23]. Figure 3a represents spectrum occupancy distributions for all frequency bands and time instants. In addition, the spectrum occupancy correlations were calculated for each frequency point. These results are plotted in Figure 3b. We note here that in these figures, each 10 MHz band belongs to a different operator.

The correlations in both time and frequency are shown in Figure 3a where the color bar shows the state of spectrum occupancy. More specifically, the vertical (horizontal) lines represent the correlation in time (frequency). Correspondingly, the correlation coefficient is demonstrated by the block-correlation pattern in Figure 3b. As seen in the figure, this correlation is high in the neighboring frequency bands of each operator. Therefore, the existence of strong correlations across both time and frequency can be observed for each operator individually.

**Figure 4:**
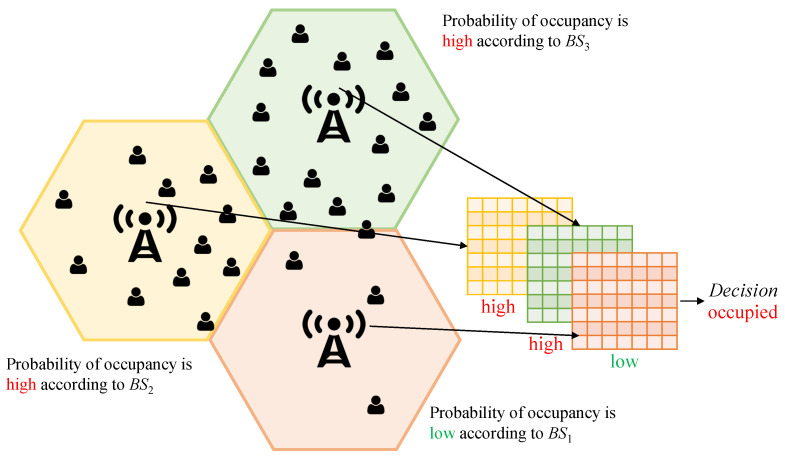
An illustrative motivation for space correlation.

In addition to the time and frequency correlations, the exploitation of spatial correlation is advantageous for the spectrum occupancy prediction problem, as illustrated in Figure 4. Here, $BS_{1}$ (main BS) is the BS that the spectrum occupancy prediction will be made on. It can be seen from this figure that when $BS_{1}$ is trained without any prior information from $BS_{2}$ and $BS_{3}$ (neighboring BSs), it decides on the future spectrum as non-occupied. However, $BS_{2}$ and $BS_{3}$ are full, and the user of $BS_{2}$ and $BS_{3}$ can occupy the spectrum of the $BS_{1}$ for the upcoming intervals, so the number of occupied bands will increase for $BS_{1}$. Exploiting the information from this dimension, spectrum occupancy can be predicted more reliably.

Motivated by the aforementioned existence of a multidimensional correlation, current literature uses tensor-based methods for spectrum occupancy prediction. However, these methods exhibit high degrees of computational complexity. Alternatively, a divide-and-conquer approach can be used. This decentralized approach divides the original problem into smaller sub-problems and solves each one individually. Then, it integrates the solutions to obtain an eventual holistic solution. The following experiment is conducted to investigate the validity of this idea. A dataset is collected according to the measurement setup for the Taksim area as detailed in Section 4. Then, the networks are trained with 2D and 3D datasets. We note here that hyperparameters of these networks are detailed in Section 5. Finally, a complexity comparison is made in terms of training and testing execution times. The results are listed in Table 1. This table shows that the computational complexity of the 3D-based method is much higher than the 2D-based method.

### 3.2. The Proposed Method

The proposed method uses a learning-based strategy. This strategy consists of two stages, which are referred to as training and testing. In the training stage, the dataset is collected and DL models are configured and trained. Then, in the testing stage, the spectrum occupancy prediction is performed. These stages are detailed below.

In the training stage, a set of spectrum measurements for training are collected by several BSs. Then, the binary occupancies of these measurements are acquired with the comparison of the measured received signal strength indicator (RSSI) with a specific threshold. We here note that more details about threshold selection are given in Section 4.1. After collecting the measurements, the dataset is obtained as follows. A sample of input is formed by a sliding window that sweeps the time and frequency (for instance, if the length of the sliding window is set to 7, then each input will have a 7 × 7 matrix of binary occupancies; this matrix will sweep time and frequency axes). Moreover, the output data of this input is the RSSI value in the medial frequency value for the upcoming time interval, which is to be predicted. According to this setting, RSSI measurements are stored in a 2D shape referring to time and frequency as an input dataset for each BS. Furthermore, their corresponding occupancies of the main BS are stored as an output dataset.

**Figure 5:**
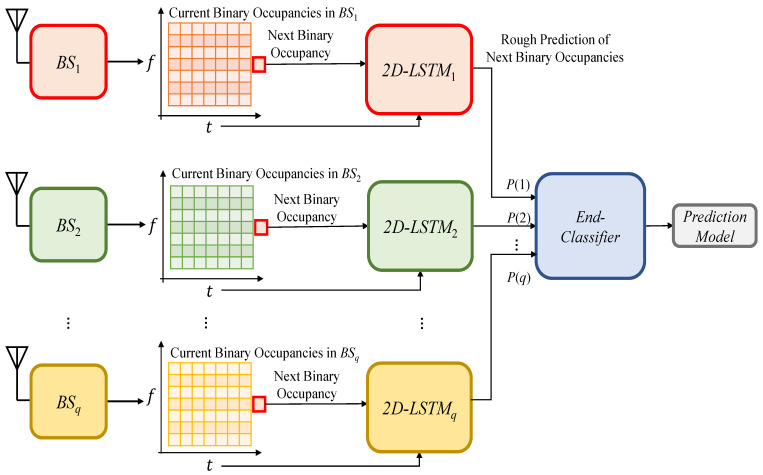
The proposed method-training stage.

Once the training data are obtained, the DL model is configured to have a total number of *q* models, where *q* denotes the number of spatially-correlated BSs, and each model accepting as input a time-frequency occupancy grid. As mentioned earlier, this configuration simultaneously incorporates space correlation and time-frequency correlations. More specifically, each LSTM model is trained over a set of pairs of a given time-frequency occupancy grid and the corresponding ground-truth occupancy status of the main BS. Then, each DL model calculates a probability of occupancy (*P*). The occupancy probabilities of all *q* models are augmented to form an occupancy probability feature vector. Afterward, the occupancy probability vector, along with the true occupancy state, form the training data pair of the end-classifier to incorporate space correlation. Finally, the end-classifier is trained over this data pair. These processes are presented in Figure 5.

**Figure 6:**
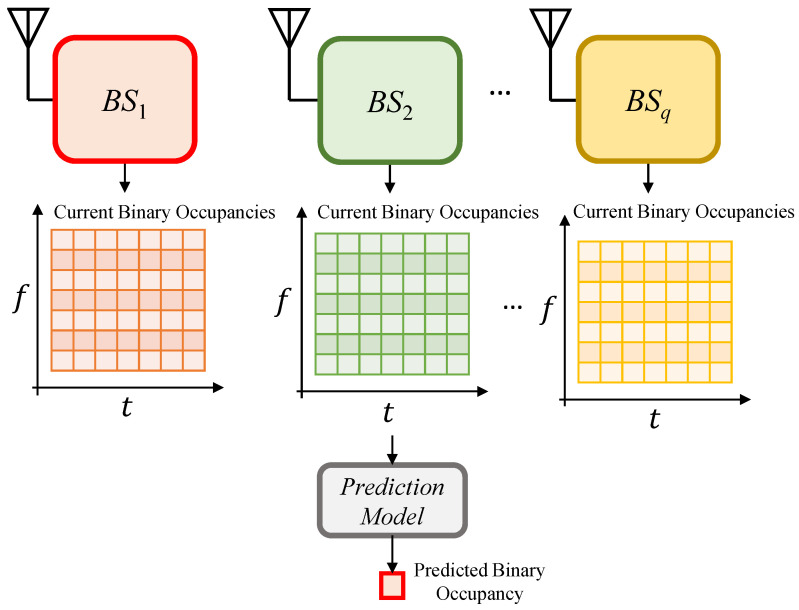
The proposed method-testing stage.

The run-time operation of the proposed method is represented by the testing stage. In this stage, spectrum measurements for time, frequency, and space lags are used to predict the corresponding spectrum occupancy probability in the upcoming time instant. This is achieved by feeding the binary occupancies of the 2D dataset as a grid to the DL models already trained, which generate the occupancy as numeric values (probabilities). Then, analogous to the setting in the training stage, the predicted *q* grid probabilities are augmented in the shape of an occupancy probability vector. Finally, the predicted occupancy probability vector is fed to the end-classifier to yield the eventual occupancy prediction. Figure 6 illustrates these processes.

### 3.3. A Note on Computational Complexity

The computational complexity of the proposed method can be roughly quantified in terms of the required execution time for training and testing stages. For the training stage, the time computational complexity can be approximated as

(10) $$T\left(n\right)=O_{l}+O_{s},$$

where $O_{l}$ and $O_{s}$ represent time complexity of a 2D-LSTM model and an end-classifier, respectively. It is noted that although composite LSTM models (*q* number of LSTM models) are used in the proposed method, their total execution times equals an LSTM model execution time since they are independent and can work in parallel.

The total number of parameters, $p_{l}$, in a standard LSTM network, including one cell in each memory block, neglecting the biases, is as follows [38]

(11) $$p_{l}=4n_{c}n_{c}+4n_{i}n_{c}+n_{c}n_{o}+3n_{c},$$

where $n_{i}$, $n_{o}$, and $n_{c}$ represent the number of input units, output units, and memory cells, respectively.

The computational complexity of LSTM models per weight is O(1) in the training stage [39]. Thus, training computational complexity per time step is $O(p_{l})$, and the total complexity of the LSTM models is $O(p_{l})$.

As an end-classifier, let us consider a two-layer neural network. The number of parameters for this classifier, $p_{s}$, is lk+ml, where *k*, *l*, and *m* denotes the number of neurons at the input, hidden, and output layers, respectively. The computational complexity of an end-classifier ($O_{s}$) can be negligible since the number of parameters is very small compared to LSTM. Consequently, the overall complexity of the training stage is $O(qp_{l})$. Furthermore, the computational complexity of training per sample is approximately two times more as compared to the complexity of testing per sample [40].

## 4. Dataset Generation

### 4.1. Measurement Setup

The measurements of the uplink (UL) private frequency band belong to one of the leading mobile network operators in Turkey and were collected by directly measuring the RSSI of the spectrum as a function of time, frequency, and space. Afterward, the binary occupancies were obtained by thresholding the RSSI measurements, where “0” represents a hole, and “1” represents the occupied spectrum. The BSs measure noise and interference power on the traffic channels (physical UL shared channel). The minimum measurable value by the BSs is −121 dBm [41]. On the other hand, a margin of 3 dB is considered to account for any variations and unforeseen effects [42,43]. Therefore, −118 dBm is set as a threshold overall in the paper. In a certain frequency, if the measured RSSI is above this adopted threshold, then this band is considered as occupied and if it is below the threshold, then this band is considered as a hole.

The measurement is conducted on a physical resource block (PRB) basis. This means that each measurement addresses 180 kHz. The accumulated noise and interference power of each PRB is averaged during the recording period. The time resolution of the recording period is 15 min. The Comba ODI-065R17M18JJJ-G receiving antenna [44] measures received signals between 852–862 MHz, where the frequency-division duplex is considered as an operational mode. We here note that two neighboring BSs are selected to exploit space correlations. While choosing these neighboring BSs, the number of handover attempts in a one-week period prior to the time of measurement was taken into consideration (the BSs that have made the highest number of handover attempts with the main BSs between 31 July 2020, and 7 August 2020, were selected as neighboring BSs). We note here that channel quality between the BS and the user; and spatial distance between the BS and the user can be used to select the best neighboring BSs as well. However, we use the number of handovers since the number of handovers is beneficial as it reflects not only channel quality (power) but also the motion pattern of the users, which was motivated in Figure 4. The numbers of handover attempts of two BSs where the most handover attempts are made with the main BSs are given in Table 2.

### 4.2. Measurement Procedure and Geographical Locations

The measurements were taken in two regions in Istanbul, Turkey, with varying levels of traffic, to investigate the generalizability of the proposed method. These areas are detailed further below.

#### 4.2.1. City Center (Taksim)

**Figure 7:**
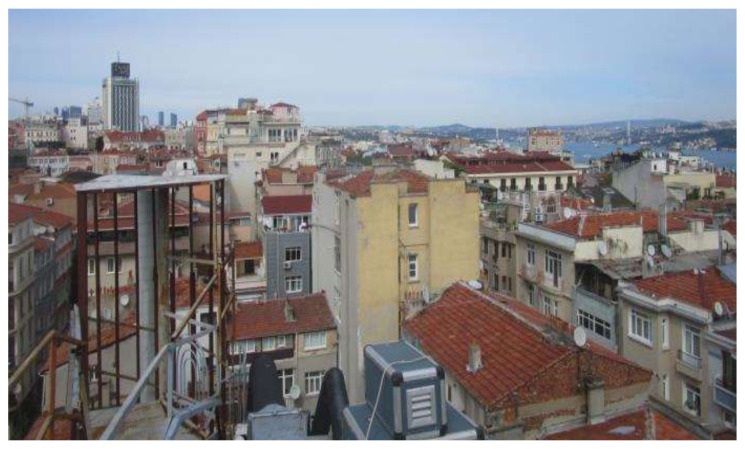
Picture of the environment for Taksim.

**Figure 8:**
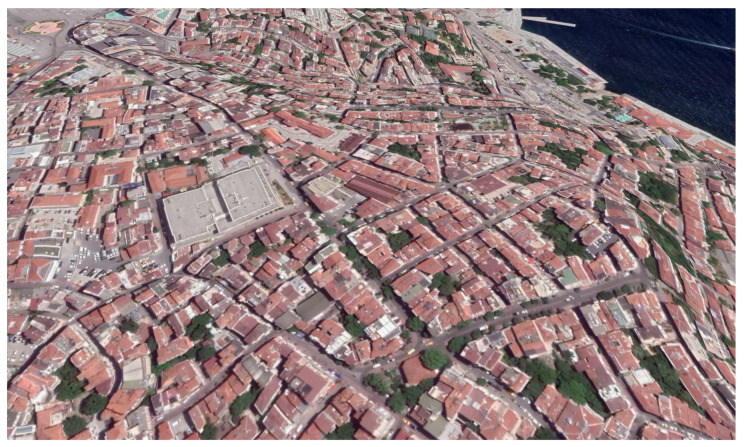
Satellite capture of the environment for Taksim.

For the measurements with high user activity, data were collected simultaneously for the whole spectrum at Taksim Square, Istanbul. The measurements were started at midnight local Istanbul time (GMT+3) on 7 August 2020, and ended at midnight on 14 August 2020. It is worth mentioning that Taksim has one of the most crowded streets in the city, with contiguous buildings around. Taksim is a flat street within a high altitude overlooking high-rise areas in general. Also, it has a central metro station, and it is an important point for transportation. That makes the area very crowded around commute times and rush hours. Furthermore, there are many dining places that make it crowded also at lunch and dinner times. Figure 7 shows a picture of the environment for the Taksim area, which has been taken from the corresponding BS. In addition, satellite captures of the area are shown in Figure 8 to show the area with a wider perspective.

#### 4.2.2. Rural Area (Silivri)

**Figure 9:**
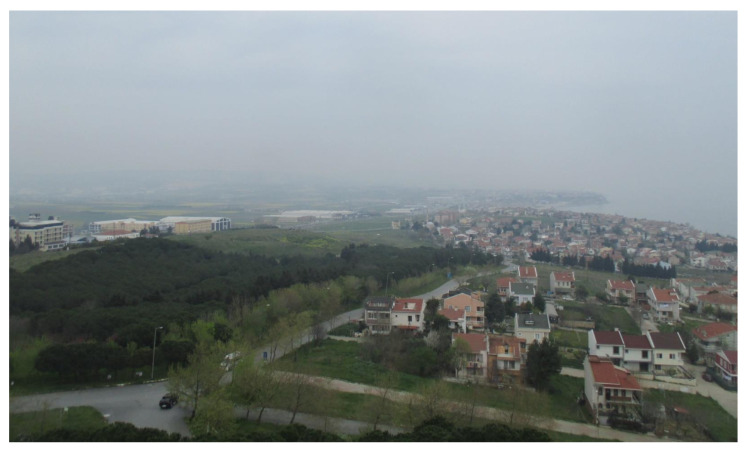
Picture of the environment for Silivri.

**Figure 10:**
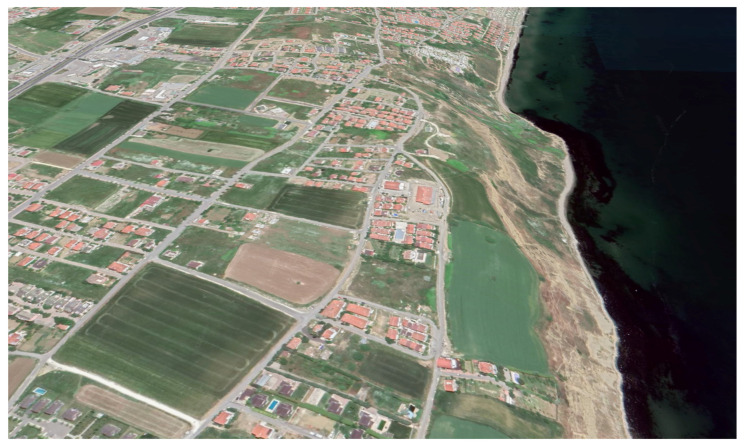
Satellite capture of the environment for Silivri.

For the measurements with low user activity, data were collected at Silivri where it is located in the rural parts of Istanbul. The measurements were made during the same time as the measurements taken at Taksim. The area is not as crowded as the city center. It has slightly wavy hills with a height of about 60 m at most. Figure 9 shows a picture of the environment for the Silivri area that has been taken from the corresponding BS. Also, a satellite capture picture is given in Figure 10.

## 5. Parameter Settings and Experimental Results

The experimental setup used is given in Section 4. A 180 kHz frequency resolution of the frequency bands within 852–862 MHz was used for the dataset. We here note that 20 kHz was used as a guard band between the subbands [45]. More details regarding the signals used for the datasets is given in Table 3. The frequency bands were measured every fifteen minutes for one week. Therefore, 201,600 points were measured in total. The binary occupancies were assigned for the related frequencies according to the threshold. A lag value was empirically set to 7 for time and frequency correlations and set to 3 for space correlation according to the performance and generalization capability of the prediction models. The first five-day dataset was used for training, the next day dataset for validation, and the remaining for testing in all of the experiments. In other words, approximately the first 71.5% of the dataset was used for training, the next 14.25% for validation, and the remaining 14.25% for testing in all of the experiments.

### 5.1. Hyperparameters of Deep Learning Models

**Figure 11:**
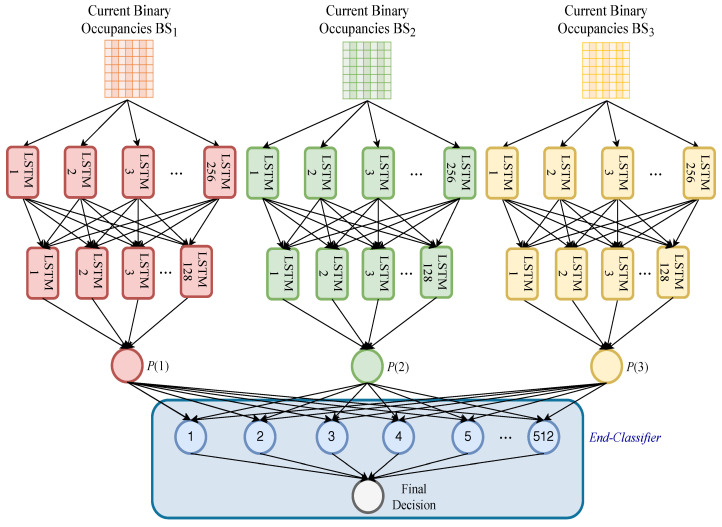
Composite two-dimensional (2D)-long short-term memory (2D-LSTMs) in spectrum occupancy prediction.

All of the DL models for spectrum occupancy predictions were implemented by Keras [46], an open-source ML library under the Python environment. All of the models were trained and tested on an MSI computer with Intel® Core™ i7-7700HQ central processing unit (CPU) @ 2.80 GHz CPU, 16 GB RAM, GeForce GTX 1050 Ti graphical processing unit, and Windows 10 operating system. All parameters of the DL models were empirically set with the consideration of the performance and generalization capability of these models. DL models were individually trained for Taksim and Silivri datasets. However, the same hyperparameters was used for both since the effect of hyperparameters were negligible for scenarios. Three types of DL models; 1D-LSTM, ConvLSTM, and 2D-LSTM models, and an end-classifier are briefly described below. We note that three 2D-LSTM models (composite 2D-LSTM models) and an end-classifier are used in the proposed method. The overall model that is used for the proposed method is illustrated in Figure 11.

- *1D-LSTM*: This model uses two LSTM hidden layers and an output layer. Particularly, 256 and 128 hidden units were used in the first and second hidden layers, respectively. The rectified linear unit (ReLU) was used as activation functions. Afterward, the probability of the occupancies was calculated in the output layer, which uses a sigmoid activation function with 1 unit. We note that one unit is enough to represent the occupancies since there are only two classes (spectrum is occupied “1” or not “0”). In total, 461,441 parameters were used. Finally, the model was trained with a batch size of 256 and 18 epochs. Efficient adaptive moment estimation (ADAM) was used with an optimum learning rate of 0.0001 during the training. Also, the logarithmic loss function was used for binary classification.
- *ConvLSTM*: The ConvLSTM model was used with 3D data as the state-of-the-art method. The model includes two ConvLSTM layers, a flatten layer, and an output layer. In the first and second ConvLSTM layers, 256 and 128 units were used, respectively. Afterward, a flatten layer was used to prepare a vector for the output layer. Finally, an output layer WAS used with one unit. In the output layer, the sigmoid function was used. In total, 4,142,721 parameters were used. A batch size of 256 and 15 epochs were used to train the model. ADAM was used for the adaptive learning rate optimization with an optimum learning rate of 0.00005. Furthermore, the logarithmic loss function was used for binary classification.
- *2D-LSTM*: This model uses two LSTM hidden layers and an output layer. More specifically, with ReLU activation functions 256 and 128 hidden units were used in the first and second LSTM hidden layers, respectively. Afterward, an output layer was used to calculate the probability of the occupancy. The sigmoid function was used in the output layer. In total, 467,585 parameters were used. Finally, the DL model was trained with a batch size of 256 and 15 epochs. ADAM was used for adaptive learning rate optimization and the optimum learning rate in this model was found at 0.00005. Again, for binary classification, the logarithmic loss function was employed.
- *An end-classifier*: A standard two-layer feed-forward network [47] was used as an end-classifier. This classifier consists of a hidden layer and an output layer. The sigmoid functions were used as activation functions. The MATLAB Neural-Network-Toolbox “nprtool” [47] was used for implementation. Scaled conjugate gradient (trainsscg), and cross-entropy (crossentropy) were used for training and performance metrics, respectively. The number of hidden neurons was set to 512 while the number of output neurons was set to one. Therefore, 25,600 parameters were used in total.

### 5.2. Performance Evaluation and Discussion

Six spectrum occupancy prediction methods are compared. These prediction methods are ARM, BIF, 1D-LSTM using only time correlation, 2D-LSTM using time and frequency correlations, ConvLSTM using multidimensions as a tensor, and the proposed method using multidimensions.

The performance of a classifier model can be evaluated in terms of *precision* (π), *recall* (ψ), and $F_{1}$-score performance metrics. The quantification of the percentage of positive results that are actually positive is measured by the *precision* metric, the percentage information of true positives that are identified correctly as positive are quantified by the *recall*, and the overall measures for the accuracy of classifier models are given by the $F_{1}$-score since it gives the harmonic average of *precision* and *recall*. These metrics are defined as follows

(12) $$\pi =\frac{\xi }{\xi +\upsilon },\psi =\frac{\xi }{\xi +\mu },F_{1}-\mathrm{score}=2\times \frac{\pi \times \psi }{\pi +\psi },$$

where ξ, υ, and μ represent the numbers of true positive, false positive, and false negative, respectively.

The results can be seen in Table 4 and Table 5. These tables show that the proposed method is superior to ARM, BIF, 1D-LSTM, and 2D-LSTM-based methods, and its performance is close to the ConvLSTM method. This is consistently true in terms of all quality metrics. Furthermore, the general performance (performance of all methods) is better in the Taksim scenario since most of the bands are occupied and so the algorithm has a stronger tendency to predict the bands as occupied.

The complexity performance analysis based on the execution times of the training and testing stages is presented in Table 6 and Table 7. We note that the value of testing complexity is given for the total computation complexity of all of the testing samples. It is evident that the proposed method has smaller execution times and faster convergence in both training and testing stages.

**Figure 12:**
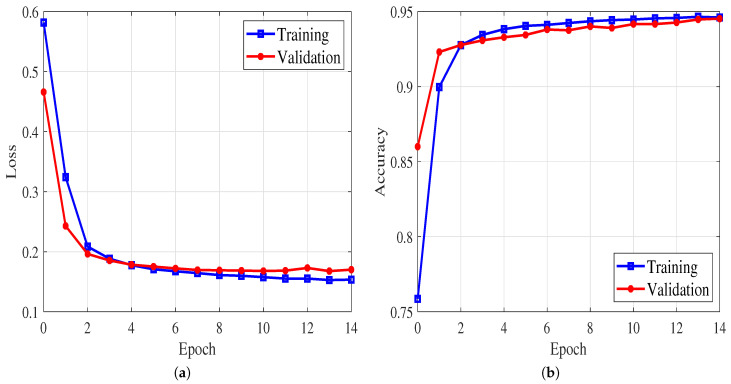
(**a**) Loss and (**b**) accuracy graphs for 2D-LSTM.

The generalization capability of an ML model is an important success criterion; from an ML perspective, a trained model should not memorize the training samples. The quality of a trained ML classifier model is shown in Figure 12 as in the training and testing losses and accuracies versus epochs for the spectrum occupancy prediction when the Taksim dataset is used with the 2D-LSTM. The figure clearly shows that the accuracy of both training and test sets converge to similar values. These results validate the generalizability of the proposed model, as there is no overfitting observed. It is worth mentioning that loss and accuracy graphs are provided only for one 2D-LSTM to prevent repetition. Similar behavior is observed for the graphs of other models and end-classifiers.

## 6. Conclusions

This paper has demonstrated the advantage of exploiting occupancy correlation over time, frequency, and space, for spectrum occupancy prediction. Three-dimensional exploitation was achieved using composite 2D-LSTM models that incorporate previous time and frequency spectrum measurements to predict the following spectrum occupancy. It has been shown that the accuracy of the 2D model can be improved by using the 3D model. Also, the proposed method provides additional accuracy over the 2D model without incurring a substantial increase in complexity. Furthermore, only a simple re-training for the end-classifier is required when data are not completely available in the proposed method. Extensive experimental results revealed performance improvements over classical prediction methods and DL models that use time and joint time/frequency correlations. On the other hand, the performance of the proposed method is close to the tensor-based method, but at a less computational cost. These results were validated in terms of the *precision*, *recall*, and $F_{1}$-score performance metrics over real-world spectrum measurements. In addition, we conducted complexity analyses in terms of training and testing execution times. The reduction in computational complexity that is achieved by the proposed method is demonstrated by these analyses. Moreover, this paper has also shown the practicality of proposed DL-based and other well known spectral occupancy prediction methods when applied to real-world measurements.

## Figures and Tables

**Table 1:** Training and testing execution time (seconds) comparison of the tensor and 2D-based method.

Method	Execution Time (s)	
	Training	Testing
The tensor-based method	608	2.8
2D-LSTM-based method	57.8	0.7

**Table 2:** The number of handover attempts between the main base station (BS) and neighboring BSs in one week.

BS	Area	
	Taksim	Silivri
$BS_{2}$	6429	3178
$BS_{3}$	4442	3138

**Table 3:** Details of the signals used for the datasets.

3GPP Band	Bandwidth	Frequency	Duplex	Technology
B20	10 MHz	852–862 Mhz (UL)	Frequency division duplexing	Long term evolution-Advanced Pro (4.5 G)

**Table 4:** The *precision* (π), *recall* (ψ), and $F_{1}$-score spectrum occupancy performances of the autoregressive model (ARM), Bayesian inference (BIF), 1D-LSTM, 2D-LSTM, ConvLSTM, and composite 2D-LSTMs (the proposed method) for the Taksim scenario.

Method	Measure		
	π	ψ	$F_{1}$-Score
ARM	0.9210	0.9681	0.9440
BIF	0.9264	0.9738	0.9495
1D-LSTM	0.9602	0.9780	0.9690
2D-LSTM	0.9720	0.9732	0.9726
ConvLSTM	0.9760	0.9763	0.9762
Composite 2D-LSTMs	0.9727	0.9742	0.9735

**Table 5:** The *precision* (π), *recall* (ψ), and $F_{1}$-score spectrum occupancy performances of ARM, BIF, 1D-LSTM, 2D-LSTM, ConvLSTM, and composite 2D-LSTMs (the proposed method) for the Silivri scenario.

Method	Measure		
	π	ψ	$F_{1}$-Score
ARM	0.8863	0.8704	0.8783
BIF	0.9336	0.9130	0.9232
1D-LSTM	0.9465	0.9165	0.9312
2D-LSTM	0.9462	0.9216	0.9338
ConvLSTM	0.9479	0.9298	0.9388
Composite 2D-LSTMs	0.9476	0.9233	0.9353

**Table 6:** Complexity analyses of ConvLSTM and the proposed method for the Taksim scenario.

Method	Execution Time (s)	
	Training	Testing
ConvLSTM	608.7	2.7
Composite 2D-LSTMs	58.1	0.7

**Table 7:** Complexity analyses of ConvLSTM and the proposed method for the Silivri scenario.

Method	Execution Time (s)	
	Training	Testing
ConvLSTM	610.3	2.9
Composite 2D-LSTMs	58.9	0.8

## Data Availability

The data presented in this study are available on request from the corresponding author. The data are not publicly available due to mobile network operator policies.

## Abbreviations

The following abbreviations are used in this manuscript:

1D	one-dimensional
2D	two-dimensional
3D	three-dimensional
5G	fifth-generation
ADAM	adaptive moment estimation
ARM	autoregressive model
BIF	Bayesian inference
BS	base station
CNN	convolutional neural networks
ConvLSTM	convolutional long short-term memory
CPU	central processing unit
CR	cognitive radio
DL	deep learning
LSTM	long-short term memory
ML	machine learning
PRB	physical resource block basis
PUs	primary users
ReLU	rectified linear unit
RSSI	received signal strength indicator
SB	subband
SUs	secondary users
UL	uplink
